# The Many Faces of *Xenopus*: *Xenopus laevis* as a Model System to Study Wolf–Hirschhorn Syndrome

**DOI:** 10.3389/fphys.2019.00817

**Published:** 2019-06-26

**Authors:** Micaela Lasser, Benjamin Pratt, Connor Monahan, Seung Woo Kim, Laura Anne Lowery

**Affiliations:** Department of Biology, Boston College, Chestnut Hill, MA, United States

**Keywords:** Wolf–Hirschhorn syndrome, *Xenopus*, development, craniofacial, neural crest cells

## Abstract

Wolf–Hirschhorn syndrome (WHS) is a rare developmental disorder characterized by intellectual disability and various physical malformations including craniofacial, skeletal, and cardiac defects. These phenotypes, as they involve structures that are derived from the cranial neural crest, suggest that WHS may be associated with abnormalities in neural crest cell (NCC) migration. This syndrome is linked with assorted mutations on the short arm of chromosome 4, most notably the microdeletion of a critical genomic region containing several candidate genes. However, the function of these genes during embryonic development, as well as the cellular and molecular mechanisms underlying the disorder, are still unknown. The model organism *Xenopus laevis* offers a number of advantages for studying WHS. With the *Xenopus* genome sequenced, genetic manipulation strategies can be readily designed in order to alter the dosage of the WHS candidate genes. Moreover, a variety of assays are available for use in *Xenopus* to examine how manipulation of WHS genes leads to changes in the development of tissue and organ systems affected in WHS. In this review article, we highlight the benefits of using *X. laevis* as a model system for studying human genetic disorders of development, with a focus on WHS.

## Introduction

Wolf–Hirschhorn syndrome (WHS) is a developmental disorder characterized by craniofacial malformations, intellectual disability, microcephaly, seizures, growth retardation, and developmental delays, though the severity of these symptoms varies from patient to patient (Fisch et al., 2010; Hannes et al., 2010; Sheth et al., 2012; Battaglia et al., 2015; Rutherford and Lowery, 2016). The core phenotype of WHS is the “Greek warrior helmet” appearance, defined by a prominent forehead, widely spaced eyes (hypertelorism), and an unusually wide and protrusive nasal bridge (Engbers et al., 2009). Other craniofacial abnormalities include an undersized jaw (micrognathia), a short philtrum, and cleft lip (Engbers et al., 2009). Most patients present with microcephaly and, less frequently, with skeletal defects, renal and urogenital defects, heart defects, hearing loss, and ocular irregularities (Andersen et al., 2014; Paradowska-Stolarz, 2014).

**FIGURE 1:**
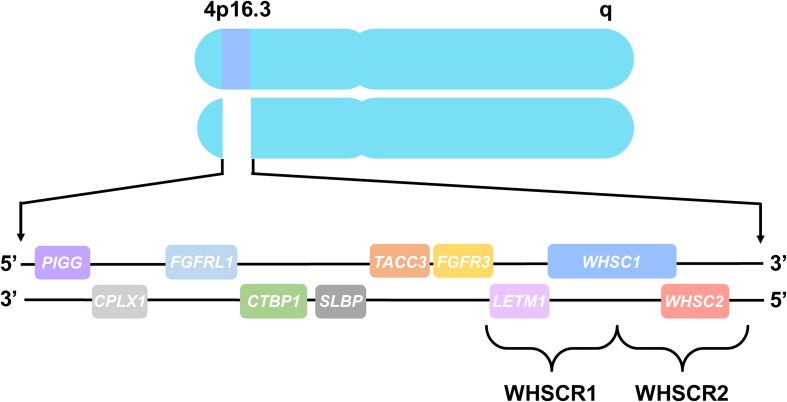
Chromosome 4 and WHS-associated genes. All genes are represented in their order from left to right, telomeric to centromeric orientation, located on the distal arm of chromosome 4p16.3. Brackets delineate WHS critical region 1 (WHSCR1), comprised of *LETM1* and *WHSC1*, and WHS critical region 2 (WHSCR2), comprised of *WHSC1* and *WHSC2*. Genes that are also mutated in WHS patients consist of *PIGG*, *CPLX1*, *FGFRL1*, *CTBP1*, *SLBP*, *TACC3*, and *FGFR3*, which flank the WHS critical regions.

The chromosomal basis of WHS has been linked to a telomeric heterozygous microdeletion on the short arm of chromosome 4, though the nature and size of this deletion differs between patients (Zollino et al., 2014; Battaglia et al., 2015; Bi et al., 2016). The central phenotypes of WHS are most strongly associated with the deletion of two adjacent, non-overlapping critical regions at 4p16.3, WHS critical region 1 (WHSCR1) and WHS critical region 2 (WHSCR2) (Battaglia et al., 2015). These regions are comprised of the primary candidate genes WHSC1, WHSC2, and LETM1 (Figure 1; Battaglia et al., 2015; Derar et al., 2018). However, most WHS patients harbor mutations that extend beyond these critical regions, affecting several flanking genes such as TACC3, FGFR3, SLBP, CTBP1, CPLX1, PIGG, MSX1, and FGFRL1, demonstrating that deletions encompassing these genes may also contribute to the presentation of WHS symptoms (Hannes et al., 2010; Endele et al., 2011; Battaglia et al., 2015; Ho et al., 2016; Rutherford and Lowery, 2016).

Due to the clinical and genetic variability of WHS, the pathology underlying the disorder has been difficult to determine. However, the characteristic facial and cardiac phenotypes of WHS suggest a particular cell population may be affected by mutations of WHS-associated genes. Vertebrate facial features are derived from or influenced by neural crest cells (NCCs), which are a group of multipotent stem cells that are born along the neural tube and migrate long distances toward their final destination (Mayor and Theveneau, 2013). Additionally, some WHS-associated genes have defined roles in epigenetic modifications or signaling pathways that are integral for proper embryonic development and cell-motility related processes (Rutherford and Lowery, 2016). Therefore, it is possible that some of the developmental defects associated with WHS may be due to abnormalities in signaling pathways that affect NCC motility or migration.

While some animal models of WHS do exist, most of these studies have focused on the cellular and molecular functions of singular WHS-associated genes, specifically WHSC1 and LETM1 (Battaglia et al., 2015). However, the link between haploinsufficiency of most WHS-associated genes to specific vertebrate developmental processes such as brain, craniofacial, and heart development, has not been carefully examined. Furthermore, clinical studies of WHS patients have provided clear evidence that the syndrome is multigenic (Battaglia et al., 2015); yet, we still lack a mechanistic understanding of how the depletion of WHS-associated genes combinatorially contributes to the phenotypic spectrum of the disorder.

The model organism, *Xenopus laevis*, has been used extensively in the research community for decades to examine fundamental developmental and cellular biological processes, making it an ideal system for investigating human genetic disorders, such as WHS. *X. laevis* offers a number of benefits, as they are inexpensive and easy to culture, maintain, manipulate, and image, compared to other vertebrates (Erdogan et al., 2016; Slater et al., 2017). Thus, *Xenopus* is well-suited to examine the role of WHS-associated genes during vertebrate development, and can be used to test whether abnormal NCC migration may be one mechanism by which mutations of these genes contribute to phenotypes of the disease.

In this review, we provide a brief overview of the known functions of WHS-associated genes and how they may be linked to developmental processes, such as NCC migration. We also highlight how *Xenopus* is an advantageous system to study WHS as a multigenic disorder, and we discuss ways in which it can be used to investigate how WHS-associated genes individually and combinatorially affect both proper embryonic development and the phenotypes linked to WHS.

## WHS Critical Region and Associated Candidate Genes

Wolf–Hirschhorn syndrome is a contiguous gene syndrome associated with small heterozygous deletions of the 4p chromosomal region. Following the initial characterization of the disorder in the 1960s, research has focused on generating a consensus of the core WHS phenotype, which includes intellectual disability, growth delay, seizures, and the typical craniofacial dysmorphisms, as well as defining the critical genomic region that gives rise to this core phenotype (Battaglia et al., 2015). The first critical region to be described, WHSCR1, consisted of a small 165 kb region encompassing two candidate genes, WHS Candidates 1 and 2 (WHSC1, WHSC2), and is deleted in all traditional cases of the disorder (Battaglia et al., 2015).

However, further clinical studies of children with WHS detected an even larger 1.9 Mb deletion that extends distally beyond WHSCR1, sufficient to produce the full WHS core phenotype (Battaglia et al., 2015). This newly described region is referred to as WHSCR2 and encompasses a more telomeric region of WHSC1, as well as a new candidate gene, leucine zipper and EF-hand containing transmembrane protein 1 (LETM1) (Battaglia et al., 2015). Moreover, many WHS patients harbor mutations of genes that flank these critical regions, providing increasing evidence that the manifestation of the disorder is not due to a single gene, but rather due to the haploinsufficiency of several closely linked genes (Battaglia et al., 2015; Rutherford and Lowery, 2016). Thus, WHS is largely considered to be a true multigenic disorder. In the subsequent sections, we describe the current known functions of the primary WHS candidate genes, as well as other WHS-associated genes, their potential roles during embryonic development, and how they may contribute to the spectrum of WHS phenotypes.

### WHSC1

WHSC1 is a histone methyltransferase, belonging to a family of nuclear receptor SET domain (NSD) proteins that possess a SET domain encoding lysine melthyltransferase activity (Nimura et al., 2009; Ezponda et al., 2013; Liu et al., 2015). Specifically, WHSC1 is associated with trimethylation of histone H3K36, a mark that is highly correlated with active transcription. WHSC1 is partially or fully deleted in nearly all cases of WHS and is thought to be responsible for many of the core WHS phenotypes, including growth delay and facial irregularities (Andersen et al., 2014; Battaglia et al., 2015; Boczek et al., 2018; Derar et al., 2018); yet, the mechanism behind which its depletion leads to these phenotypes remains to be elucidated.

The spatial and temporal expression of WHSC1, as well as its transcriptional regulation activity, suggests that it plays an important role during development (Rutherford and Lowery, 2016). WHSC1 is a known regulator of TWIST, a key player in both the epithelial-to-mesenchymal transition (EMT) and cell migration (Ezponda et al., 2013). Moreover, WHSC1 is expressed in the developing nervous system, pharyngeal arches, and frontal facial region in both mice and *Xenopus laevis*, as well as jaw and heart in mice (Stec et al., 1998; Nimura et al., 2009; Yamada-Okabe et al., 2010; Liu et al., 2015; Mills et al., 2019), which is significant, given that many of these structures are both derived from the neural crest and are affected in WHS.

However, until recently, WHSC1 function had not been directly associated with NCC migration. To gain further insight into the role of WHSC1, specifically during processes that govern craniofacial morphogenesis, Mills et al. (2019) sought to observe the effects of reducing WHSC1 dosage levels in *Xenopus laevis* during embryonic development. They found that WHSC1 KD results in reduced length and area of the pharyngeal arches, as well as reduced NCC migration speed. Depletion of WHSC1 also causes severe craniofacial and brain morphology defects including widening of the facial width and area, and reduction of forebrain size. Together, these results are the first to demonstrate that reduced levels of WHSC1 cause aberrant NCC migration, which may lead to some of the craniofacial abnormalities seen in WHS patients. These results are consistent with other studies showing that WHSC1 depletion leads to irregularities in brain, cartilage, and bone formation, specifically incomplete motor neuron development in zebrafish, deficiencies in midline fusion and cleft palate in mice, as well as delayed bone ossification in both zebrafish and mice (Nimura et al., 2009; Yamada-Okabe et al., 2010; Ezponda et al., 2013; Liu et al., 2015), indicating a conserved developmental role for WHSC1 among various species.

An important question still remains as to which signaling pathways are affected by WHSC1 reduction and how they contribute to abnormal NCC migratory patterns and WHS phenotypes. A promising possibility is the Wnt signaling pathway, which has already been connected to WHSC1 dysregulation in the context of cancer (Toyokawa et al., 2011). Toyokawa et al. (2011) suggests that WHSC1 dosage may impact canonical Wnt signaling by affecting nuclear levels of beta-catenin through transcriptional regulation. Moreover, in *Xenopus*, canonical Wnt signaling has been shown to be important for NCC specification and migration during development, as well as subsequent craniofacial morphogenesis (Maj et al., 2016; Li et al., 2018). Thus, further experiments are necessary to examine whether alterations in WHSC1 dosage impact the Wnt signaling pathway, potentially leading to abnormal NCC migration and the spectrum of defects observed in WHS.

### WHSC2

The WHSC2 gene, also known as NELF-A, encodes a component of the negative elongation factor complex, which negatively regulates RNA polymerase II progression during elongation, subsequently altering the expression of its target genes (Kerzendorfer et al., 2012). Though WHSC2 is commonly deleted in most WHS patients, it has not emerged as a primary contributor to any specific WHS phenotype (Battaglia et al., 2015). However, the cellular and molecular roles of WHSC2 have begun to be illuminated and functionally linked to other WHS-affected genes.

Depletion of WHSC2 in both cell culture and in *Drosophila* has been shown to reduce cellular proliferation and lead to irregularities in cell cycle progression (Gilchrist et al., 2008; Yung et al., 2009). Interestingly in breast cancer cell lines, reduction of the NELF-E component of the NELF complex is associated with lower levels of trimethylated H3K36 on promoters of NELF-regulated genes (Sun and Li, 2010). As previously mentioned, levels of trimethylated H3K36 are influenced by the activity of WHSC1, highlighting a potential link between WHSC1 and WHSC2 (NELF-A) in altering gene expression, although this remains to be examined.

WHSC2 also functions to recruit stem-loop binding protein (SLBP) to the 3′ ends of histone pre-mRNAs (Narita et al., 2007). SLBP lies telomeric to WHSC2 and mutations of this gene have been found in some WHS patients, though the functional impact of SLBP haploinsufficiency is unclear. SLBP is critical for the correct processing of histone pre-mRNAs and regulating histone degradation. Histone biogenesis is essential for epigenetic gene regulation through histone post-translational modifications and maintenance of chromatin structure (Margueron and Reinberg, 2010). Thus, it is possible that there is a functional connection between WHSC1, WHSC2, and SLBP, and reduced activity of these genes may have impacts on histone levels and transcriptional regulation, which could adversely affect normal development in the context of WHS.

Until recently, WHSC2 function during vertebrate tissue and organ development had not been carefully studied. Mills et al. (2019) found that WHSC2 expression in *Xenopus* is enriched in the developing nervous system, pharyngeal arches, and frontal facial region. Additionally, altered levels of WHSC2 lead to abnormalities in craniofacial, cartilage, and brain development. Specifically, reduction of WHSC2 causes smaller facial width and area, smaller cartilage elements, as well as smaller brain size (Mills et al., 2019), indicating that WHSC2 may play a larger role regarding WHS phenotypes than what was previously thought. It is possible that these phenotypes arise as a consequence of decreased cellular proliferation, as WHSC2 has already been linked to this process (Sun and Li, 2010), though this connection still needs to be tested. Moreover, it would be interesting to examine whether reduced dosage levels of WHSC2 in combination with WHSC1 or SLBP enhances these phenotypes, providing further evidence that these genes are functionally linked to one another.

### LETM1

LETM1 is localized to the inner mitochondrial membrane and is important for maintaining normal mitochondrial function by participating in Ca^2+^/H^+^ exchange and regulating Ca^2+^ homeostasis within the matrix (Dimmer et al., 2008; Jiang et al., 2013). LETM1 has been proposed as the candidate gene for seizures in WHS patients (Endele et al., 1999; McQuibban et al., 2010; Andersen et al., 2014; Battaglia et al., 2015). Heterozygous deletion of LETM1 in mice led to altered glucose metabolism and impaired control of ATP levels in the brain, which could explain the increased susceptibility to seizures observed in these animals (Jiang et al., 2013). However, some WHS patients with LETM1 deletions lack seizures and conversely, some WHS patients have seizures, yet have no detectable mutations in LETM1 (Battaglia et al., 2015). Therefore, it is likely that LETM1 is not solely responsible for the occurrence of seizures and that deletion of additional genes are required for the full expression of the phenotype.

In *Xenopus*, LETM1 is expressed ubiquitously during early embryonic development. As development progresses, more defined expression is observed in the pharyngeal arches, craniofacial and brain regions (Mills et al., 2019). Reduced dosage of LETM1 in *Xenopus* revealed that it is critical for proper craniofacial morphology and may contribute to some of the facial defects associated with WHS, which had not been previously demonstrated (Mills et al., 2019). Interestingly, knocking down LETM1 did not have a significant effect on NCC migration or motility, which is surprising, given the robust craniofacial phenotype observed. However, it is possible that LETM1 may play an entirely different role in these cells. Proper mitochondrial function and regulation of energy metabolism is critical for determining cell fate and maintaining cell survival (Khacho et al., 2019). Therefore, while reduced dosage of LETM1 may not directly affect NCC migration, it may greatly impact the metabolic homeostasis of NCCs and lead to downstream effects on differentiation, proliferation, or apoptosis during craniofacial morphogenesis. As LETM1 depletion in fibroblasts derived from WHS patients has already been associated with decreased cellular proliferation (Doonan et al., 2014), this seems a promising avenue of future research to understand the precise molecular mechanisms of LETM1 involvement in the pathogenesis of WHS.

### Additional WHS-Associated Genes

While all WHS patients harbor full or partial deletions of the WHSCRs, most have mutations that affect multiple genes flanking these regions. It is widely accepted that haploinsufficiency of the WHS critical region genes alone cannot account for the full spectrum of WHS phenotypes (Battaglia et al., 2015). Thus, it is likely that these flanking genes, including TACC3, FGFR3, FGFRL1, SLBP, CTBP1, CPLX1, and PIGG, and MSX1 also contribute to WHS in some capacity.

Fibroblast growth factor receptor 3 (FGFR3) and fibroblast growth factor receptor like 1 (FGFRL1) are both involved in signaling pathways that influence cell division and differentiation. Both FGFR3 and FGFRL1 have been proposed as candidate genes responsible for the skeletal and craniofacial malformations of WHS patients (Battaglia et al., 2015). Homozygous null mouse lines for Fgfrl1 recapitulate many WHS phenotypes such as hypoplasia of skeletal elements, delayed fusion and bone ossification, abnormal forebrain development, and congenital heart defects (CHD) (Catela et al., 2009). Conversely, Fgfr3 null mice display severe kyphosis, abnormally long tails and femurs, which is indicative of prolonged bone growth (Simon and Bergemann, 2008). With opposing phenotypes, it is possible that these two genes antagonize each other to regulate chondrocyte proliferation and may genetically interact to control bone growth. Moreover, FGFR3 has been shown to directly affect NCC migration in chick, with its loss leading to slower NCC migration velocity and increased neural crest death close to the neural tube (Sato et al., 2011). However, it remains unclear whether the loss of either FGFR3 or FGFRL1 is directly linked to abnormal NCC migration in the WHS phenotypes.

Transforming acidic coiled-coil protein 3 (TACC3) is a microtubule plus-end tracking (+TIP) protein that resides on the end of polymerizing microtubules and has been shown to regulate microtubule dynamics during neural development (Nwagbara et al., 2014; Bearce et al., 2015; Cammarata et al., 2016; Erdogan et al., 2017). The ways in which TACC3 dosage levels affect gross morphological phenotypes reflected in WHS patients was recently examined and provides a strong link to abnormal NCC migration. In *Xenopus*, TACC3 expression is enriched in the pharyngeal arches, as well as the frontal brain and facial regions (Mills et al., 2019). Knockdown of TACC3 led to overt craniofacial and cartilage defects, including smaller facial width, area and angle, which are likely due to overall smaller cartilage elements (Mills et al., 2019). Furthermore, *in vivo* migration of NCCs was affected as a consequence of reduced TACC3 levels. This manipulation also severely affected forebrain development, reflecting the microcephaly phenotype commonly associated with WHS. This is interesting given that TACC3 is also known to be an essential centrosome adaptor that is critical for proper cell division, and almost all microcephaly associated genetic defects involve proteins that maintain centrosomes and regulate the mitotic spindle (Peset and Vernos, 2008; Lasser et al., 2018). Thus, it is likely that TACC3 contributes to some WHS phenotypes, possibly by adversely altering the mitotic spindle such that it has downstream effects that influence cell fate and cell migration.

It has been suggested that C-terminal binding protein 1 (CTBP1), complexin 1 (CPLX1), and phosphatidylinositol glycan anchor biosynthesis class G (PIGG) are possible candidates for developmental delay, intellectual disabilities, and seizures in WHS patients (Zollino et al., 2014). CTBP1 is a transcriptional co-repressor known to mediate E-cadherin repression and play a key role in EMT (Zhang et al., 2013). Increased Ctbp activity has been shown to reduce epileptic behavior in rats following a ketogenic diet by forcing energy production via the TCA cycle and oxidation of NADH (Garriga-Canut et al., 2006). Reduced levels of NADH stimulates Ctbp activity and could be a mechanism by which loss of CTBP1 contributes to seizures. Ctbp1 mutant mice also exhibit defects in skeletal and muscle development (Hildebrand and Soriano, 2002). As CTBP1 is known to be involved in EMT (Zhang et al., 2013), it seems plausible that mutations of this gene may result in these phenotypes by influencing NCC migration.

CPLX1 is a cytosolic protein that is important for synaptic vesicle exocytosis and regulation of neurotransmitter release (Glynn et al., 2005). Mutations of CPLX1 have been associated with epilepsy and intellectual disability, and Cplx1 knockout mice exhibit pronounced motor and social deficits (Glynn et al., 2005; Drew et al., 2007; Redler et al., 2017). Pathogenic variants of PIGG are associated with intellectual disability, hypotonia, and seizures (Makrythanasis et al., 2016). PIGG localizes to the endoplasmic reticulum and is responsible for the biogenesis of GPI anchor proteins in zebrafish, which is critical for expression of voltage-gated sodium channels in a subset of neurons (Nakano et al., 2010). However, it is clear that further research is needed to determine how these genes are distinctly contributing to the development of organs and tissues that are affected in WHS.

MSX1 (Msh homeobox 1) functions as a transcriptional repressor during embryogenesis and has been suggested as a candidate gene for the oral and dental malformations seen in some WHS patients (Nieminen et al., 2003). Mutations of MSX1 have been associated with non-syndromic cleft palate and tooth agenesis in humans, and Msx1-deficient mice exhibit severe craniofacial abnormalities, cleft palate, and absence of teeth (Satokata and Maas, 1994; Zhang et al., 2002; Nieminen et al., 2003). In *Xenopus*, MSX1 has been shown to be critical for NCC induction and regulates the expression of genes that are necessary for proper NCC migration (Monsoro-Burq et al., 2005; Bonano et al., 2008; Macrì et al., 2016). Thus, it is likely that haploinsufficiency of MSX1 contributes to some craniofacial and dental defects associated with WHS by affecting NCC induction, as well as having downstream regulatory effects on genes that influence their subsequent migration.

## Using *Xenopus Laevis* as a Model to Study WHS

In an effort to answer some outstanding questions regarding the pathogenesis of WHS, it is critical to choose a model organism that is ideal for studying human genetic diseases. Every model system has its benefits and limitations; however, *X. laevis* has emerged as a particularly advantageous model for studying human genetic diseases, like WHS. While mouse models are an excellent mammalian system due to the similarity between the mouse and human genomes and the large genetic toolkit available, they are very costly to house and maintain. Moreover, litter sizes are small, embryonic development occurs *in utero*, and creating genetic lines that harbor mutant alleles is time-consuming. While zebrafish produce many offspring and have well-developed genetic manipulation strategies, their genome has lost a great deal of synteny with mammals and many relevant disease-related genes do not perform the same function (Garcia de la Serrana et al., 2014). Zebrafish also lack organ systems, such as limbs, digits, and lungs, that are involved in many human congenital syndromes. Additionally, the zebrafish heart only has one atrium and one ventricle, and cannot fully model developmental heart abnormalities, like those associated with WHS.

However, using *Xenopus* as a system to investigate human genetic diseases of development has enormous potential, and can complement more established systems, like mouse or zebrafish, to enhance our knowledge about the function of understudied genes and the underlying mechanisms by which developmental abnormalities arise. In the following sections, we highlight why *X. laevis* is an advantageous model to choose for investigating developmental disorders, with a specific focus on how it can be used to examine multiple aspects of WHS.

### Advantages of *Xenopus laevis* as a Model Organism

*Xenopus* is an excellent system for relatively high throughput analysis of genetic manipulation on vertebrate embryonic development. *Xenopus* shares a high degree of synteny with humans and a majority of disease-associated genes are conserved between these species (Hellsten et al., 2010; Session et al., 2016). Despite *X. laevis* having a duplicated genome, genetic manipulation strategies can still be utilized. However, *X. tropicalis* may be more suitable, depending on the number of genes being studied, as they are diploid, making genetic manipulation much easier and more rapid. The *Xenopus* genome is widely available to the research community through the efforts of *Xenbase*, an online resource that has organized current annotated genetic information, protocols, *Xenopus* anatomy and development, scientific literature, and provides useful sequencing tools, such as the genome browser and *Xenopus* specific BLAST (James-Zorn et al., 2018).

A key characteristic of the *Xenopus* model is the ease of acquiring large amounts of high quality embryos by inducing females to lay eggs via hormone priming (Sive et al., 2007a, b; Erdogan et al., 2016; Slater et al., 2017). Hundreds of embryos can be obtained in a single clutch, enabling numerous embryos to be manipulated and observed in a single experiment. *Xenopus* development occurs rapidly and externally, with gastrulation and neurulation occurring between 9 and 26 h post fertilization, and organogenesis almost complete by 5 days post fertilization (Nieuwkoop and Faber, 1994; Zahn et al., 2017). *Xenopus* organ development and morphology have been well-characterized and is comparable to those of mammalian systems, including orofacial (Dickinson, 2016), heart (Hempel and Kühl, 2016), kidney (Getwan and Lienkamp, 2017), and nervous system development (Pratt and Khakhalin, 2013; Lee-Liu et al., 2017), all of which are affected in WHS patients. Moreover, *Xenopus* is being used extensively as a model to understand a number of different human genetic diseases that lead to defects in these systems, such as congenital heart disorders (Boskovski et al., 2013; Sojka et al., 2014; Duncan and Khokha, 2016; Deniz et al., 2017), kidney disease (Lienkamp, 2016), ciliopathies (Brooks and Wallingford, 2015; Wallmeier et al., 2016), orofacial defects (Tahir et al., 2014; Dickinson, 2016), and neurodevelopmental disorders (Pratt and Khakhalin, 2013; Tandon et al., 2017; Willsey et al., 2018).

**FIGURE 2:**
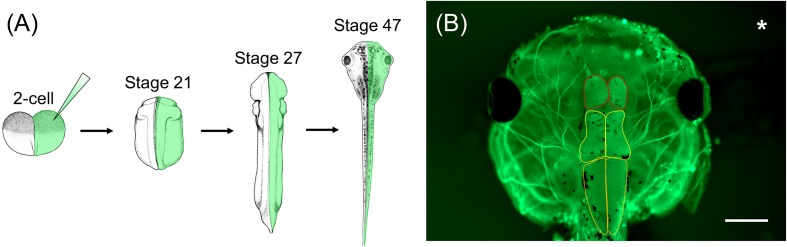
*Xenopus* one-sided injections allow for side-by-side comparison to wild-type gene expression following genetic manipulation. **(A)** *Xenopus* embryos can be injected in one cell at the 2-cell stage with genetic manipulation macromolecules and fluorescent mRNA. At stage 21, embryos can be sorted depending on the side that was injected (left vs. right) and used for various assays throughout development. **(B)** Representative image of stage 47 *X. laevis* tadpole immunolabeled for acetylated tubulin. Asterisk represents the side that was injected with MO and fluorescent mRNA. Brain morphology, such as forebrain size (red outline), midbrain size (yellow outline), and hindbrain size (orange outline), of the manipulated side can be compared to the unaltered, wild-type side. Scale bar: 500 μm.

In order to characterize WHS-associated gene functions in relation to development and disease, *Xenopus* embryos can be injected with a variety of materials to manipulate gene expression, such as CRISPR/cas9 or morpholino oligonucleotides (MOs), in either the whole embryo or selected blastomeres (up to the 64-cell stage) (Mimoto and Christian, 2011; Tandon et al., 2012, 2017; Bestman and Cline, 2014; Bhattacharya et al., 2015; Wang et al., 2015; DeLay et al., 2016; Feehan et al., 2017; Moody, 2018a, b). As the lineage of individual cells has been well-documented, injections can be precisely targeted to specific tissues and organs that are affected in WHS, such as the heart, kidney, or brain. A unique feature of *Xenopus* compared to other models is the ability to perform one-sided embryo injections, wherein only one side of the embryo is experimentally manipulated and the opposite side serves as an internal control (Figure 2; Willsey et al., 2018; Mills et al., 2019). Thus, assessing phenotypic consequences that arise as a result of genetic manipulation can be compared side-by-side to wild-type gene expression.

Morpholino oligonucleotides are particularly useful in modeling genetic diseases, like WHS, as they can be easily titrated to reduce gene dosage levels similar to that in human patients (Tahir et al., 2014; Blum et al., 2015; McCammon and Sive, 2015). Because WHS-associated mutations result in haploinsufficiency, a full knockout of the candidate genes would not appropriately model the disease, and simultaneous knockdown of genes can be achieved by injecting multiple MOs at once, allowing for concurrent knockdown of genes that are often deleted together in WHS (Blum et al., 2015). While it is certainly possible to produce mouse lines with mutations in multiple genes (Simon and Bergemann, 2008), this is a costlier and more time-consuming process than the equivalent in *X. laevis.* As with all manipulation strategies, the appropriate controls must be used to account for off-target effects, such as generating more than one MO, dose dependency, and rescuing phenotypes by co-injecting mRNA that is not targeted by the MO (Blum et al., 2015; Gentsch et al., 2018).

The CRISPR/cas9 system has also been used as an extremely effective method to knock out target genes in both *X. tropicalis* and *X. laevis* (Bhattacharya et al., 2015; Wang et al., 2015; Tandon et al., 2017). While the off-target effects of CRISPR/cas9 are generally minimal, the use of proper controls is critical by carefully designing multiple sgRNAs and performing rescue experiments to confirm that any phenotypes observed are due to the knockout of a particular gene (Nakayama et al., 2013; Wang et al., 2015). The CRISPR/cas9 system can be employed to validate phenotypes that arise as a result of MO-mediated gene knockdown by comparing phenotypes generated by both methods (Bhattacharya et al., 2015; Willsey et al., 2018). Overall, *Xenopus* is an excellent system for investigating developmental disorders, such as WHS, and for elucidating the mechanism by which manipulation of WHS-associated genes alters proper embryonic development.

### *Xenopus laevis* as a Model for Studying Neural Crest Cell Migration

As the underlying cellular and molecular mechanisms that cause WHS are unknown, it is essential to investigate the basis behind the disorder. One particularly promising avenue of research in this regard is the migration of the NCC population in the developing embryo (Rutherford and Lowery, 2016). As stated previously, NCCs are a multipotent stem cell population that originate along the neural tube, delaminate, and migrate throughout the developing embryo to their final destinations. Once at their proper locations, NCCs differentiate and contribute to various tissues and organ systems, including craniofacial cartilage and bone, smooth muscle of the heart, peripheral and enteric neurons, melanocytes, and glia (Bronner and LeDouarin, 2012). Considering craniofacial abnormalities are one of the defining phenotypes of WHS, the link to potential aberrations in NCC migration is strong. Moreover, almost all WHS-associated genes are connected with NCC migration or signaling pathways that may influence NCC differentiation or proliferation in some capacity. Therefore, research into NCC migration in relation to WHS is of great interest.

**FIGURE 3:**
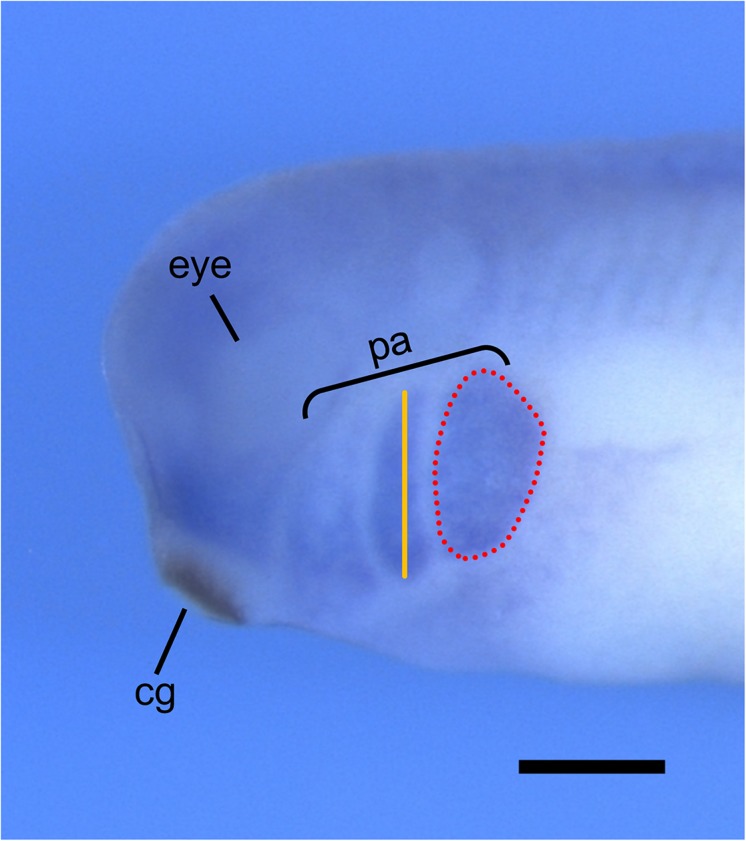
Analysis of *in vivo* NCC migration using whole-mount *in situ* hybridization. Representative image of stage 30 *X. laevis* embryo labeled for xTWIST, a marker for NCCs. Measurements of the length (orange line) and area (red dotted line) of each individual pharyngeal arch can be done using ImageJ. Pharyngeal arch length is measured from the most dorsal point to the most ventral point of each individual arch. Pharyngeal arch area is measured as the area around the periphery of each individual arch. cg, cement gland; pa, pharyngeal arches. Scale bar: 500 μm.

*Xenopus laevis* is an ideal model organism for studying NCC migration, as both *in vivo* and *in vitro* NCC migration can be tracked through multiple methods. NCC migration can be observed *in vivo* by performing transplantation assays, whereby NCCs are dissected from GFP-injected *Xenopus* embryos and transplanted to wild-type host embryos (Borchers et al., 2000; Cousin, 2018). These embryos can then be imaged using time-lapse confocal microscopy and NCC migration can be analyzed by measuring the number, width, and migration distance of the GFP-marked cranial segments. Additionally, two transgenic *X. laevis* frog lines, PAX3-GFP and SOX10-GFP, are available through the National *Xenopus* Resource (NXR) to study the *in vivo* development and migration of NCCs (Alkobtawi et al., 2018). *In vivo* NCC migration can also be analyzed through whole-mount *in situ* hybridization by observing the morphology of the pharyngeal arches using a marker for NCCs, such as TWIST or SOX9 (Figure 3; Devotta et al., 2016; Szabó et al., 2016).

Neural crest cell motility can be examined *in vitro* by dissecting NCCs from stage 16 *Xenopus* embryos and culturing explants on fibronectin-coated coverslips (Cousin and Alfandari, 2018). Migratory behavior of NCC explants can be observed using time-lapse confocal microscopy and multiple parameters of migration can be measured including velocity and dispersion (DeSimone et al., 2005; Milet and Monsoro-Burq, 2014; Cousin and Alfandari, 2018). Moreover, chemotaxis assays can be performed *in vitro* to assess the directional migration of NCCs toward an external, soluble factor by coating beads with a chemoattractant cue, such as SDF-1 (Theveneau and Mayor, 2011; Theveneau et al., 2013; Shellard and Mayor, 2016; Szabó et al., 2016). Recently, these methods were successfully used in *X. laevis* to test whether knockdown of particular WHS genes led to changes in NCC migration both *in vivo* and *in vitro* (Mills et al., 2019), demonstrating how *X. laevis* can provide a unique and varied approach to study how alterations in NCC migratory behavior may contribute to phenotypes of the disease.

### *Xenopus laevis* as a Model for Studying Other Aspects of WHS

Craniofacial abnormalities are one of the defining phenotypes of WHS and *X. laevis* has emerged as an excellent system for determining which WHS-associated genes are important for craniofacial morphogenesis. As orofacial development is highly conserved between *Xenopus* and other mammalian species, craniofacial abnormalities in *Xenopus* often resemble phenotypes present in human patients (Dickinson and Sive, 2007, 2006; Tahir et al., 2014; Dubey and Saint-Jeannet, 2017). Various techniques to assess changes in *Xenopus* orofacial development have already been developed and used to study craniofacial defects associated with human genetic disorders (Tahir et al., 2014; Gonzalez Malagon et al., 2018; Mills et al., 2019). Similar to NCC transplant assays, facial transplant assays can be performed in *Xenopus* (Jacox L. A. et al., 2014; Jacox L. et al., 2014; Jacox et al., 2016; Gonzalez Malagon et al., 2018). This technique allows for the examination of gene or protein function in particular cell types involved in facial development, eliminating non-specific effects in the whole embryo, and can be used to study signaling mechanisms or mechanical forces that are involved in orofacial development (Dickinson, 2016).

**FIGURE 4:**
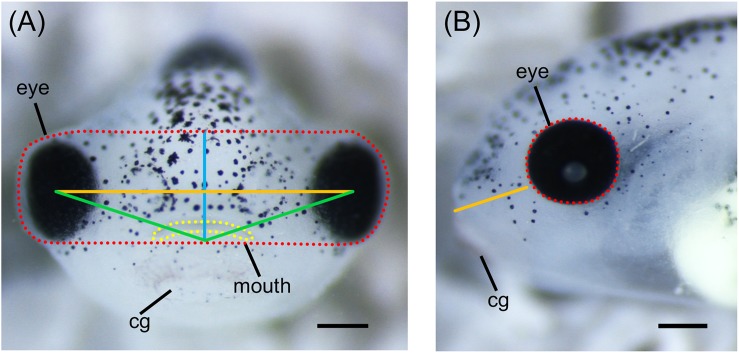
Measurements of *Xenopus* craniofacial features. **(A)** Representative frontal view image of stage 42 *X. laevis* tadpole. Measurements of facial features include facial height (blue line), facial width (orange line), facial area (red dotted line), facial angle (green line), and mouth roundness (yellow dotted line). Facial height is measured from the top of the middle of the eyes to the top of the cement gland. Facial width is measured from the middle of one eye to the middle of the opposite eye. Facial area is measured as the area around the top of the eyes to the top of the cement gland. Facial angle is measured as the angle between the middle of the eyes and the top of the cement gland. Mouth roundness is measured as the area around the periphery of the mouth. **(B)** Representative lateral view image of stage 42 *X. laevis* tadpole. Measurements of facial features include eye area (red dotted line) and snout length (orange line). Eye area is measured as the area around the periphery of the eye. Snout length is measured from the most anterior part of the face to the bottom of the eye. All measurements can be performed using ImageJ. cg, cement gland. Scale bar: 100 μm.

**FIGURE 5:**
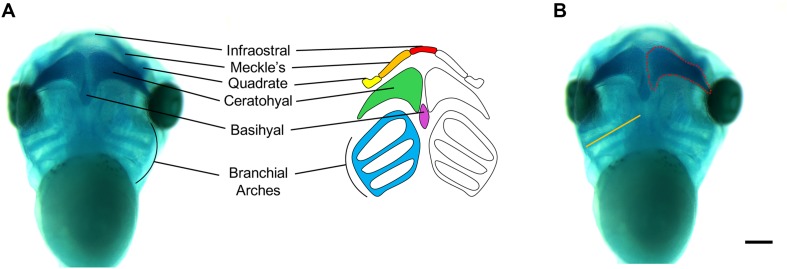
Measurements of *Xenopus* cartilage elements. **(A)** *Xenopus* cartilage anatomy with all major cartilage elements labeled in various colors. **(B)** Representative ventral view image of stage 45 *X. laevis* tadpole stained for Alcian blue. Measurements of cartilage elements include branchial arch length (orange line) and ceratohyal area (red dotted line), which can be performed using ImageJ. Scale bar: 300 μm.

More robust quantitative methods to examine alterations of orofacial development have also been adapted for use in *Xenopus*. Measurements of various craniofacial features such as facial width, height, angle, and area can be done on embryos at different developmental stages using ImageJ (Figure 4; Kennedy and Dickinson, 2014a; Tahir et al., 2014). These measurements can be combined with geometric morphometrics in order to detect subtle differences in face shape and size throughout development (Kennedy and Dickinson, 2014b; Dickinson, 2016). Furthermore, techniques for visualizing cartilage morphology, such as Alcian blue staining, can be used to determine whether craniofacial abnormalities arise as a result of defects in cartilage development (Figure 5; Tahir et al., 2014; Devotta et al., 2016).

It is important to note that there are some craniofacial and cartilage morphological differences between *Xenopus* and humans that will prevent some direct correlations to disease pathology. For example, Meckel’s cartilage will eventually become a component of the lower jaw in both *Xenopus* and humans, however in humans, it will also become part of the middle ear skeletal structures. Nevertheless, using these techniques in *Xenopus* has provided evidence that some WHS-associated genes are required for proper craniofacial development and contribute to the signature craniofacial dysmorphisms seen in WHS patients (Mills et al., 2019).

Individuals with WHS display a variety of other symptoms such as microcephaly, heart defects, and renal defects. Whether WHS-associated genes are required for proper development of these organ and tissue systems in relation to WHS still remains unknown. However, many techniques exist in *Xenopus* to study brain, heart, and kidney development. *Xenopus* neurodevelopment has been explored extensively using both *in vitro* and *in vivo* methods (Erdogan et al., 2016; Slater et al., 2017). Due to the large size of its growth cones, *X. laevis* is an excellent model to study cytoskeletal dynamics in axon outgrowth and guidance during early development. Moreover, live imaging can be done on both retinal and spinal axonal tracts *in vivo* (Erdogan et al., 2016; Slater et al., 2017). Additionally, changes in brain morphology can be quantified simply by measuring the area of the forebrain, midbrain, and hindbrain (Figure 2B), and used as a technique to reflect the microcephaly phenotype associated with WHS (Willsey et al., 2018; Mills et al., 2019).

Congenital heart defects have emerged as the most life-threatening birth defect in newborn patients across different developmental disorders, including WHS (Duncan and Khokha, 2016; Garfinkel and Khokha, 2017). Early heart development is highly conserved between *Xenopus* and mammalian systems, and assays to analyze CHD candidate genes have been created for use in *Xenopus* (Sojka et al., 2014; Duncan and Khokha, 2016; Hempel and Kühl, 2016; Deniz et al., 2017; Garfinkel and Khokha, 2017; Tandon et al., 2017). The effect of genetic manipulation on heart development can be explored at early tadpole stages using whole-mount *in situ* hybridization, immunohistochemistry with anti-tropomyosin (Tmy) antibody, scanning electron microscopy (SEM), transmission electron microscopy (TEM), or optical coherence tomography (OCT) (Sojka et al., 2014; Hempel and Kühl, 2016; Deniz et al., 2017). These assays help to visualize and detect improperly looped hearts, failed chamber formation, and abnormal structure of the adjacent connective tissue, and can be used to elucidate how mutations of WHS genes lead to CHD (Sojka et al., 2014; Duncan and Khokha, 2016).

*In vitro* explant, transplant, and ablation techniques can be used to derive insight into how mutations of genes associated with renal disease affect the progression of kidney development in *Xenopus* (Getwan and Lienkamp, 2017). *In vivo* experiments of kidney development range from optogenetic manipulations of physiological parameters (calcium, pH) to characterizations of electrophysiological recordings (Lienkamp, 2016; Getwan and Lienkamp, 2017). Together, *X. laevis* can be used as a powerful system to study how mutations of WHS-associated genes affect the development of multiple tissue and organ systems.

## Conclusion

*Xenopus laevis* has emerged as an effective model organism for studying human genetic disorders of development, such as WHS. *Xenopus* embryos can be collected in large numbers and are easy to maintain, manipulate, and image. Genetic manipulation strategies are straightforward, allow for the concurrent KD or KO of multiple genes at once, and can be titrated to reduce gene dosage levels similar to that in human patients with WHS. Numerous techniques exist in *Xenopus* to study a variety of tissue and organ systems in order to understand the function of WHS-associated genes during embryonic development. Most importantly, *Xenopus* provides a system in which phenotypes of WHS, including craniofacial abnormalities, brain defects, heart defects, and kidney defects can be easily examined. Moreover, the mechanism by which mutations of WHS-associated genes affects embryonic development can be studied in *Xenopus*, such as changes in NCC migration. Thus, *X. laevis* is an excellent model system for investigating the underlying pathology of WHS, as well as many other human genetic diseases.

## Author Contributions

BP and LL contributed to the conception and design of the manuscript. BP and ML wrote the first draft of the manuscript. CM and SK wrote the sections of the manuscript. LL edited the manuscript. All authors contributed to the revision, read, and approved the final version of the manuscript for submission.

## Conflict of Interest Statement

The authors declare that the research was conducted in the absence of any commercial or financial relationships that could be construed as a potential conflict of interest.

## References

[B1] Alkobtawi M. Ray H. Barriga E. H. Moreno M. Kerney R. Monsoro-Burq A.-H. (2018). Characterization of Pax3 and Sox10 transgenic *Xenopus laevis* embryos as tools to study neural crest development. *Dev. Biol*. 444 (Suppl. 1), S202–S208. 29522707 10.1016/j.ydbio.2018.02.020 PMC6453020

[B2] Andersen E. F. Carey J. C. Earl D. L. Corzo D. Suttie M. Hammond P. (2014). Deletions involving genes WHSC1 and LETM1 may be necessary, but are not sufficient to cause Wolf-Hirschhorn Syndrome. *Eur. J. Hum. Genet.* 22 464–470. 10.1038/ejhg.2013.192 23963300 PMC3953918

[B3] Battaglia A. Carey J. C. South S. T. (2015). Wolf-Hirschhorn syndrome: a review and update. *Am. J. Med. Genet. C Semin. Med. Genet.* 169 216–223. 10.1002/ajmg.c.31449 26239400

[B4] Bearce E. A. Erdogan B. Lowery L. A. (2015). TIPsy tour guides: how microtubule plus-end tracking proteins (+TIPs) facilitate axon guidance. *Front. Cell. Neurosci.* 9:241. 10.3389/fncel.2015.00241 26175669 PMC4485311

[B5] Bestman J. E. Cline H. T. (2014). Morpholino studies in Xenopus brain development. *Methods Mol. Biol.* 1082 155–171. 10.1007/978-1-62703-655-9_11 24048933

[B6] Bhattacharya D. Marfo C. A. Li D. Lane M. Khokha M. K. (2015). CRISPR/Cas9: an inexpensive, efficient loss of function tool to screen human disease genes in Xenopus. *Dev. Biol.* 408 196–204. 10.1016/j.ydbio.2015.11.003 26546975 PMC4684459

[B7] Bi W. Cheung S. W. Breman A. M. Bacino C. A. (2016). 4p16.3 microdeletions and microduplications detected by chromosomal microarray analysis: new insights into mechanisms and critical regions. *Am. J. Med. Genet. A* 170 2540–2550. 10.1002/ajmg.a.37796 27287194

[B8] Blum M. De Robertis E. M. Wallingford J. B. Niehrs C. (2015). Morpholinos: antisense and sensibility. *Dev. Cell* 35 145–149. 10.1016/j.devcel.2015.09.017 26506304

[B9] Boczek N. J. Lahner C. A. Nguyen T.-M. Ferber M. J. Hasadsri L. Thorland E. C. (2018). Developmental delay and failure to thrive associated with a loss-of-function variant in WHSC1 (n.d.). *Am. J. Med. Genet. A* 176 2798–2802. 10.1002/ajmg.a.40498 30345613

[B10] Bonano M. Tríbulo C. De Calisto J. Marchant L. Sánchez S. S. Mayor R. (2008). A new role for the Endothelin-1/Endothelin-A receptor signaling during early neural crest specification. *Dev. Biol.* 323 114–129. 10.1016/j.ydbio.2008.08.007 18775422

[B11] Borchers A. Epperlein H. H. Wedlich D. (2000). An assay system to study migratory behavior of cranial neural crest cells in *Xenopus*. *Dev. Genes Evol.* 210 217–222. 10.1007/s004270050307 11180825

[B12] Boskovski M. T. Yuan S. Pedersen N. B. Goth C. K. Makova S. Clausen H. (2013). The heterotaxy gene GALNT11 glycosylates Notch to orchestrate cilia type and laterality. *Nature* 504 456–459. 10.1038/nature12723 24226769 PMC3869867

[B13] Bronner M. E. LeDouarin N. M. (2012). Development and evolution of the neural crest: an overview. *Dev. Biol.* 366 2–9. 10.1016/j.ydbio.2011.12.042 22230617 PMC3351559

[B14] Brooks E. R. Wallingford J. B. (2015). *In vivo* investigation of cilia structure and function using *Xenopus*. *Methods Cell Biol.* 127 131–159. 10.1016/bs.mcb.2015.01.018 25837389 PMC4433029

[B15] Cammarata G. M. Bearce E. A. Lowery L. A. (2016). Cytoskeletal social networking in the growth cone: how +TIPs mediate microtubule-actin cross-linking to drive axon outgrowth and guidance. *Cytoskeleton* 73 461–476. 10.1002/cm.21272 26783725 PMC4955630

[B16] Catela C. Bilbao-Cortes D. Slonimsky E. Kratsios P. Rosenthal N. Te Welscher P. (2009). Multiple congenital malformations of Wolf-Hirschhorn syndrome are recapitulated in Fgfrl1 null mice. *Dis. Model. Mech.* 2 283–294. 10.1242/dmm.002287 19383940 PMC2675798

[B17] Cousin H. (2018). Cranial neural crest transplants. *Cold Spring Harb. Protoc.* 2018:db.rot097402. 10.1101/pdb.prot097402 29321285 PMC6016762

[B18] Cousin H. Alfandari D. (2018). Cranial neural crest explants. *Cold Spring Harb. Protoc.* 2018:db.rot097394. 10.1101/pdb.prot097394 29321283 PMC5834405

[B19] DeLay B. D. Krneta-Stankic V. Miller R. K. (2016). Technique to target microinjection to the developing *Xenopus* kidney. *J. Vis. Exp.* 111:e53799. 10.3791/53799 27168375 PMC4876824

[B20] Deniz E. Jonas S. Hooper M. N Griffin J. Choma M. A. Khokha M. K. (2017). Analysis of craniocardiac malformations in *Xenopus* using optical coherence tomography. *Sci. Rep.* 7:42506. 10.1038/srep42506 28195132 PMC5307353

[B21] Derar N. Al-Hassnan Z. N. Al-Owain M. Monies D. Abouelhoda M. Meyer B. F. (2018). De novo truncating variants in WHSC1 recapitulate the Wolf-Hirschhorn (4p16.3 microdeletion) syndrome phenotype. *Genet. Med.* 21 185–188. 10.1038/s41436-018-0014-8 29892088

[B22] DeSimone D. W. Davidson L. Marsden M. Alfandari D. (2005). The *Xenopus* embryo as a model system for studies of cell migration. *Methods Mol. Biol.* 294 235–245. 15576916 10.1385/1-59259-860-9:235

[B23] Devotta A. Juraver-Geslin H. Gonzalez J. A. Hong C.-S. Saint-Jeannet J. -P. (2016). Sf3b4-depleted *Xenopus* embryos: a model to study the pathogenesis of craniofacial defects in Nager syndrome. *Dev. Biol.* 415 371–382. 10.1016/j.ydbio.2016.02.010 26874011 PMC4914463

[B24] Dickinson A. Sive H. (2007). Positioning the extreme anterior in *Xenopus*: cement gland, primary mouth and anterior pituitary. *Semin. Cell Dev. Biol.* 18 525–533. 10.1016/j.semcdb.2007.04.002 17509913

[B25] Dickinson A. J. G. (2016). Using frogs faces to dissect the mechanisms underlying human orofacial defects. *Semin. Cell Dev. Biol.* 51 54–63. 10.1016/j.semcdb.2016.01.016 26778163 PMC4798872

[B26] Dickinson A. J. G. Sive H. (2006). Development of the primary mouth in *Xenopus laevis*. *Dev. Biol.* 295 700–713. 10.1016/j.ydbio.2006.03.054 16678148

[B27] Dimmer K. S. Navoni F. Casarin A. Trevisson E. Endele S. Winterpacht A. (2008). LETM1, deleted in Wolf-Hirschhorn syndrome is required for normal mitochondrial morphology and cellular viability. *Hum. Mol. Genet.* 17 201–214. 10.1093/hmg/ddm297 17925330

[B28] Doonan P. J. Chandramoorthy H. C. Hoffman N. E. Zhang X. Cárdenas C. Shanmughapriya S. (2014). LETM1-dependent mitochondrial Ca^2+^ flux modulates cellular bioenergetics and proliferation. *FASEB J.* 28 4936–4949. 10.1096/fj.14-256453 25077561 PMC4200331

[B29] Drew C. J. G. Kyd R. J. Morton A. J. (2007). Complexin 1 knockout mice exhibit marked deficits in social behaviours but appear to be cognitively normal. *Hum. Mol. Genet.* 16 2288–2305. 10.1093/hmg/ddm181 17652102

[B30] Dubey A. Saint-Jeannet J.-P. (2017). Modeling human craniofacial disorders in *Xenopus*. *Curr. Pathobiol. Rep.* 5 79–92. 10.1007/s40139-017-0128-8 28255527 PMC5327820

[B31] Duncan A. R. Khokha M. K. (2016). *Xenopus* as a model organism for birth defects-Congenital heart disease and heterotaxy. *Semin. Cell Dev. Biol.* 51 73–79. 10.1016/j.semcdb.2016.02.022 26910255 PMC4809202

[B32] Endele S. Fuhry M. Pak S. J. Zabel B. U. Winterpacht A. (1999). LETM1, a novel gene encoding a putative EF-hand Ca^2+^-binding protein, flanks the Wolf-Hirschhorn syndrome (WHS) critical region and is deleted in most WHS patients. *Genomics* 60 218–225. 10.1006/geno.1999.5881 10486213

[B33] Endele S. Nelkenbrecher C. Bördlein A. Schlickum S. Winterpacht A. (2011). C4ORF48, a gene from the Wolf-Hirschhorn syndrome critical region, encodes a putative neuropeptide and is expressed during neocortex and cerebellar development. *Neurogenetics* 12 155–163. 21287218 10.1007/s10048-011-0275-8

[B34] Engbers H. van der Smagt J. J. van ’t Slot R. Vermeesch J. R. Hochstenbach R. Poot M. (2009). Wolf-Hirschhorn syndrome facial dysmorphic features in a patient with a terminal 4p16.3 deletion telomeric to the WHSCR and WHSCR 2 regions. *Eur. J. Hum. Genet*. 17 129–132. 10.1038/ejhg.2008.168 18830230 PMC2985965

[B35] Erdogan B. Cammarata G. M. Lee E. J. Pratt B. C. Francl A. F. Rutherford E. L. (2017). The microtubule plus-end-tracking protein TACC3 promotes persistent axon outgrowth and mediates responses to axon guidance signals during development. *Neural Dev.* 12:3. 28202041 10.1186/s13064-017-0080-7 PMC5312526

[B36] Erdogan B. Ebbert P. T. Lowery L. A. (2016). Using *Xenopus laevis* retinal and spinal neurons to study mechanisms of axon guidance in vivo and in vitro. *Semin. Cell Dev. Biol.* 51 64–72. 10.1016/j.semcdb.2016.02.003 26853934 PMC4798887

[B37] Ezponda T. Popovic R. Shah M. Y. Martinez-Garcia E. Zheng Y. Min D.-J. (2013). The histone methyltransferase MMSET/WHSC1 activates TWIST1 to promote an epithelial-mesenchymal transition and invasive properties of prostate cancer. *Oncogene* 32 2882–2890. 10.1038/onc.2012.297 22797064 PMC3495247

[B38] Feehan J. M. Chiu C. N. Stanar P. Tam B. M. Ahmed S. N. Moritz O. L. (2017). Modeling dominant and recessive forms of retinitis pigmentosa by editing three rhodopsin-encoding genes in *Xenopus laevis* using Crispr/Cas9. *Sci. Rep.* 7:6920. 28761125 10.1038/s41598-017-07153-4 PMC5537283

[B39] Fisch G. S. Grossfeld P. Falk R. Battaglia A. Youngblom J. Simensen R. (2010). Cognitive-behavioral features of Wolf-Hirschhorn syndrome and other subtelomeric microdeletions. *Am. J. Med. Genet. C Semin. Med. Genet.* 154C 417–426. 10.1002/ajmg.c.30279 20981770

[B40] Garcia de la Serrana D. Mareco E. A. Johnston I. A. (2014). Systematic variation in the pattern of gene paralog retention between the teleost superorders Ostariophysi and Acanthopterygii. *Genome Biol. Evol.* 6 981–987. 10.1093/gbe/evu074 24732281 PMC4007551

[B41] Garfinkel A. M. Khokha M. K. (2017). An interspecies heart-to-heart: using *Xenopus* to uncover the genetic basis of congenital heart disease. *Curr. Pathobiol. Rep.* 5 187–196. 10.1007/s40139-017-0142-x 29082114 PMC5658036

[B42] Garriga-Canut M. Schoenike B. Qazi R. Bergendahl K. Daley T. J. Pfender R. M. (2006). 2-Deoxy-D-glucose reduces epilepsy progression by NRSF-CtBP-dependent metabolic regulation of chromatin structure. *Nat. Neurosci.* 9 1382–1387. 10.1038/nn1791 17041593

[B43] Gentsch G. E. Spruce T. Monteiro R. S. Owens N. D. L. Martin S. R. Smith J. C. (2018). Innate immune response and off-target mis-splicing are common morpholino-induced side effects in *Xenopus*. *Dev. Cell* 44 597–610.e10. 10.1016/j.devcel.2018.01.022 29478923 PMC5861998

[B44] Getwan M. Lienkamp S. S. (2017). Toolbox in a tadpole: *Xenopus* for kidney research. *Cell Tissue Res.* 369 143–157. 10.1007/s00441-017-2611-2 28401306

[B45] Gilchrist D. A. Nechaev S. Lee C. Ghosh S. K. B. Collins J. B. Li L. (2008). NELF-mediated stalling of Pol II can enhance gene expression by blocking promoter-proximal nucleosome assembly. *Genes Dev.* 22 1921–1933. 10.1101/gad.1643208 18628398 PMC2492738

[B46] Glynn D. Drew C. J. Reim K. Brose N. Morton A. J. (2005). Profound ataxia in complexin I knockout mice masks a complex phenotype that includes exploratory and habituation deficits. *Hum. Mol. Genet.* 14 2369–2385. 10.1093/hmg/ddi239 16000319

[B47] Gonzalez Malagon S. G. Lopez Muñoz A. M. Doro D. Bolger T. G. Poon E. Tucker E. R. (2018). Glycogen synthase kinase 3 controls migration of the neural crest lineage in mouse and *Xenopus*. *Nat. Commun.* 9:1126. 29555900 10.1038/s41467-018-03512-5 PMC5859133

[B48] Hannes F. Drozniewska M. Vermeesch J. R. Haus O. (2010). Duplication of the Wolf-Hirschhorn syndrome critical region causes neurodevelopmental delay. *Eur. J. Med. Genet.* 53 136–140. 10.1016/j.ejmg.2010.02.004 20197130

[B49] Hellsten U. Harland R. M. Gilchrist M. J. Hendrix D. Jurka J. Kapitonov V. (2010). The genome of the Western clawed frog *Xenopus tropicalis*. *Science* 328 633–636. 10.1126/science.1183670 20431018 PMC2994648

[B50] Hempel A. Kühl M. (2016). A matter of the heart: the African clawed frog *Xenopus* as a model for studying vertebrate cardiogenesis and congenital heart defects. *J. Cardiovasc. Dev. Dis.* 3:21. 10.3390/jcdd3020021 29367567 PMC5715680

[B51] Hildebrand J. D. Soriano P. (2002). Overlapping and unique roles for C-terminal binding protein 1 (CtBP1) and CtBP2 during mouse development. *Mol. Cell. Biol.* 22 5296–5307. 10.1128/MCB.22.15.5296-5307.2002 12101226 PMC133942

[B52] Ho K. S. South S. T. Lortz A. Hensel C. H. Sdano M. R. Vanzo R. J. (2016). Chromosomal microarray testing identifies a 4p terminal region associated with seizures in Wolf-Hirschhorn syndrome. *J. Med. Genet.* 53 256–263. 10.1136/jmedgenet-2015-103626 26747863 PMC4819617

[B53] Jacox L. Chen J. Rothman A. Lathrop-Marshall H. Sive H. (2016). Formation of a “Pre-mouth Array” from the extreme anterior domain is directed by neural crest and Wnt/PCP signaling. *Cell Rep.* 16 1445–1455. 10.1016/j.celrep.2016.06.073 27425611 PMC4972695

[B54] Jacox L. Sindelka R. Chen J. Rothman A. Dickinson A. Sive H. (2014). The extreme anterior domain is an essential craniofacial organizer acting through Kinin-Kallikrein signaling. *Cell Rep.* 8 596–609. 10.1016/j.celrep.2014.06.026 25043181 PMC4135435

[B55] Jacox L. A. Dickinson A. J. Sive H. (2014). Facial transplants in *Xenopus laevis* embryos. *J. Vis. Exp*. 85:e50697. 10.3791/50697 24748020 PMC4089428

[B56] James-Zorn C. Ponferrada V. Fisher M. E. Burns K. Fortriede J. Segerdell E. (2018). Navigating xenbase: an integrated *Xenopus* genomics and gene expression database. *Methods Mol. Biol.* 1757 251–305. 10.1007/978-1-4939-7737-6_10 29761462 PMC6853059

[B57] Jiang D. Zhao L. Clish C. B. Clapham D. E. (2013). Letm1, the mitochondrial Ca^2+^/H^+^ antiporter, is essential for normal glucose metabolism and alters brain function in Wolf-Hirschhorn syndrome. *Proc. Natl. Acad. Sci. U.S.A.* 110 E2249–E2254. 10.1073/pnas.1308558110 23716663 PMC3683736

[B58] Kennedy A. E. Dickinson A. J. (2014a). Quantification of orofacial phenotypes in *Xenopus*. *J. Vis. Exp*. 93:e52062. 10.3791/52062 25407252 PMC4353423

[B59] Kennedy A. E. Dickinson A. J. (2014b). Quantitative analysis of orofacial development and median clefts in *Xenopus laevis*. *Anat. Rec.* 297 834–855. 10.1002/ar.22864 24443252

[B60] Kerzendorfer C. Hannes F. Colnaghi R. Abramowicz I. Carpenter G. Vermeesch J. R. (2012). Characterizing the functional consequences of haploinsufficiency of NELF-A (WHSC2) and SLBP identifies novel cellular phenotypes in Wolf-Hirschhorn syndrome. *Hum. Mol. Genet.* 21 2181–2193. 10.1093/hmg/dds033 22328085

[B61] Khacho M. Harris R. Slack R. S. (2019). Mitochondria as central regulators of neural stem cell fate and cognitive function. *Nat. Rev. Neurosci.* 20 34–48. 10.1038/s41583-018-0091-3 30464208

[B62] Lasser M. Tiber J. Lowery L. A. (2018). The role of the microtubule cytoskeleton in neurodevelopmental disorders. *Front. Cell. Neurosci.* 12:165. 10.3389/fncel.2018.00165 29962938 PMC6010848

[B63] Lee-Liu D. Méndez-Olivos E. E. Muñoz R. Larraín J. (2017). The African clawed frog *Xenopus laevis*: a model organism to study regeneration of the central nervous system. *Neurosci. Lett.* 652 82–93. 10.1016/j.neulet.2016.09.054 27693567

[B64] Li J. Perfetto M. Neuner R. Bahudhanapati H. Christian L. Mathavan K. (2018). *Xenopus* ADAM19 regulates Wnt signaling and neural crest specification by stabilizing ADAM13. *Development* 145:dev158154. 10.1242/dev.158154 29540504 PMC5963864

[B65] Lienkamp S. S. (2016). Using *Xenopus* to study genetic kidney diseases. *Semin. Cell Dev. Biol.* 51 117–124. 10.1016/j.semcdb.2016.02.002 26851624

[B66] Liu S. Higashihori N. Yahiro K. Moriyama K. (2015). Retinoic acid inhibits histone methyltransferase Whsc1 during palatogenesis. *Biochem. Biophys. Res. Commun.* 458 525–530. 10.1016/j.bbrc.2015.01.148 25677622

[B67] Macrì S. Simula L. Pellarin I. Pegoraro S. Onorati M. Sgarra R. (2016). Hmga2 is required for neural crest cell specification in *Xenopus laevis*. *Dev. Biol.* 411 25–37. 10.1016/j.ydbio.2016.01.014 26806704

[B68] Maj E. Künneke L. Loresch E. Grund A. Melchert J. Pieler T. (2016). Controlled levels of canonical Wnt signaling are required for neural crest migration. *Dev. Biol.* 417 77–90. 10.1016/j.ydbio.2016.06.022 27341758

[B69] Makrythanasis P. Kato M. Zaki M. S. Saitsu H. Nakamura K. Santoni F. A. (2016). Pathogenic variants in PIGG cause intellectual disability with seizures and hypotonia. *Am. J. Hum. Genet.* 98 615–626. 10.1016/j.ajhg.2016.02.007 26996948 PMC4833197

[B70] Margueron R. Reinberg D. (2010). Chromatin structure and the inheritance of epigenetic information. *Nat. Rev. Genet.* 11 285–296. 10.1038/nrg2752 20300089 PMC3760772

[B71] Mayor R. Theveneau E. (2013). The neural crest. *Development* 140 2247–2251. 10.1242/dev.091751 23674598

[B72] McCammon J. M. Sive H. (2015). Addressing the genetics of human mental health disorders in model organisms. *Annu. Rev. Genomics Hum. Genet.* 16 173–197. 26002061 10.1146/annurev-genom-090314-050048

[B73] McQuibban A. G. Joza N. Megighian A. Scorzeto M. Zanini D. Reipert S. (2010). A *Drosophila* mutant of LETM1, a candidate gene for seizures in Wolf-Hirschhorn syndrome. *Hum. Mol. Genet.* 19 987–1000. 10.1093/hmg/ddp563 20026556

[B74] Milet C. Monsoro-Burq A.-H. (2014). Dissection of *Xenopus laevis* neural crest for *in vitro* explant culture or *in vivo* transplantation. *J. Vis. Exp*. 85:e51118. 10.3791/51118 24637938 PMC4123508

[B75] Mills A. Bearce E. Cella R. Kim S. W. Selig M. Lee S. (2019). Wolf-Hirschhorn Syndrome-associated genes are enriched in motile neural crest and affect craniofacial development in *Xenopus laevis*. *Front. Physiol.* 10:431 10.3389/fphys.2019.00431 PMC6474402 31031646

[B76] Mimoto M. S. Christian J. L. (2011). Manipulation of gene function in *Xenopus laevis*. *Methods Mol. Biol.* 770 55–75. 10.1007/978-1-61779-210-6_3 21805261 PMC3911881

[B77] Monsoro-Burq A. -H. Wang E. Harland R. (2005). *Msx1* and *Pax3* cooperate to mediate FGF8 and WNT signals during *Xenopus* neural crest induction. *Dev. Cell* 8 167–178. 10.1016/j.devcel.2004.12.017 15691759

[B78] Moody S. A. (2018a). Lineage tracing and fate mapping in *Xenopus* embryos. *Cold Spring Harb. Protoc.* 2018:db.rot097253. 29769388 10.1101/pdb.prot097253

[B79] Moody S. A. (2018b). Microinjection of mRNAs and oligonucleotides. *Cold Spring Harb. Protoc.* 2018:db.rot097261. 29769401 10.1101/pdb.prot097261

[B80] Nakano Y. Fujita M. Ogino K. Saint-Amant L. Kinoshita T. Oda Y. (2010). Biogenesis of GPI-anchored proteins is essential for surface expression of sodium channels in zebrafish Rohon-Beard neurons to respond to mechanosensory stimulation. *Development* 137 1689–1698. 10.1242/dev.047464 20392743

[B81] Nakayama T. Fish M. B. Fisher M. Oomen-Hajagos J. Thomsen G. H. Grainger R. M. (2013). Simple and efficient CRISPR/Cas9-mediated targeted mutagenesis in *Xenopus tropicalis*. *Genesis* 51 835–843. 10.1002/dvg.22720 24123613 PMC3947545

[B82] Narita T. Yung T. M. C. Yamamoto J. Tsuboi Y. Tanabe H. Tanaka K. (2007). NELF interacts with CBC and participates in 3’ end processing of replication-dependent histone mRNAs. *Mol. Cell* 26 349–365. 10.1016/j.molcel.2007.04.011 17499042

[B83] Nieminen P. Kotilainen J. Aalto Y. Knuutila S. Pirinen S. Thesleff I. (2003). *MSX1* gene is deleted in Wolf-Hirschhorn syndrome patients with oligodontia. *J. Dent. Res.* 82 1013–1017. 10.1177/154405910308201215 14630905

[B84] Nieuwkoop P. D. Faber J. (1994). *Normal Table of Xenopus Laevis (Daudin): A Systematical and Chronological Survey of the Development From the Fertilized Egg Till the End of Metamorphosis.* (New York, NY: Garland Pub).

[B85] Nimura K. Ura K. Shiratori H. Ikawa M. Okabe M. Schwartz R. J. (2009). A histone H3 lysine 36 trimethyltransferase links Nkx2-5 to Wolf-Hirschhorn syndrome. *Nature* 460 287–291. 10.1038/nature08086 19483677

[B86] Nwagbara B. U. Faris A. E. Bearce E. A. Erdogan B. Ebbert P. T. Evans M. F. (2014). TACC3 is a microtubule plus end-tracking protein that promotes axon elongation and also regulates microtubule plus end dynamics in multiple embryonic cell types. *Mol. Biol. Cell* 25 3350–3362. 10.1091/mbc.E14-06-1121 25187649 PMC4214782

[B87] Paradowska-Stolarz A. M. (2014). Wolf-Hirschhorn syndrome (WHS) - literature review on the features of the syndrome. *Adv. Clin. Exp. Med.* 23 485–489. 10.17219/acem/24111 24979523

[B88] Peset I. Vernos I. (2008). The TACC proteins: TACC-ling microtubule dynamics and centrosome function. *Trends Cell Biol.* 18 379–388. 10.1016/j.tcb.2008.06.005 18656360

[B89] Pratt K. G. Khakhalin A. S. (2013). Modeling human neurodevelopmental disorders in the *Xenopus* tadpole: from mechanisms to therapeutic targets. *Dis. Model. Mech.* 6 1057–1065. 10.1242/dmm.012138 23929939 PMC3759326

[B90] Redler S. Strom T. M. Wieland T. Cremer K. Engels H. Distelmaier F. (2017). Variants in CPLX1 in two families with autosomal-recessive severe infantile myoclonic epilepsy and ID. *Eur. J. Hum. Genet.* 25 889–893. 10.1038/ejhg.2017.52 28422131 PMC5520065

[B91] Rutherford E. L. Lowery L. A. (2016). Exploring the developmental mechanisms underlying Wolf-Hirschhorn Syndrome: evidence for defects in neural crest cell migration. *Dev. Biol.* 420 1–10. 10.1016/j.ydbio.2016.10.012 27777068 PMC5193094

[B92] Sato A. Scholl A. M. Kuhn E. N. Kuhn E. B. Stadt H. A. Decker J. R. (2011). FGF8 signaling is chemotactic for cardiac neural crest cells. *Dev. Biol.* 354 18–30. 10.1016/j.ydbio.2011.03.010 21419761 PMC3090535

[B93] Satokata I. Maas R. (1994). Msx1 deficient mice exhibit cleft palate and abnormalities of craniofacial and tooth development. *Nat. Genet.* 6 348–356. 10.1038/ng0494-348 7914451

[B94] Session A. M. Uno Y. Kwon T. Chapman J. A. Toyoda A. Takahashi S. (2016). Genome evolution in the allotetraploid frog *Xenopus laevis*. *Nature* 538 336–343. 10.1038/nature19840 27762356 PMC5313049

[B95] Shellard A. Mayor R. (2016). Chemotaxis during neural crest migration. *Semin. Cell Dev. Biol.* 55 111–118. 10.1016/j.semcdb.2016.01.031 26820523

[B96] Sheth F. Akinde O. R. Datar C. Adeteye O. V. Sheth J. (2012). Genotype-phenotype characterization of Wolf-Hirschhorn syndrome confirmed by FISH: case reports. *Case Rep. Genet.* 2012:878796. 10.1155/2012/878796 23227376 PMC3512217

[B97] Simon R. Bergemann A. D. (2008). Mouse models of Wolf-Hirschhorn syndrome. *Am. J. Med. Genet. C Semin. Med. Genet.* 148C 275–280. 10.1002/ajmg.c.30184 18932126

[B98] Sive H. L. Grainger R. M. Harland R. M. (2007a). Inducing ovulation in *Xenopus laevis*. *CSH Protoc.* 2007:db.rot4734. 10.1101/pdb.prot4734 21357079

[B99] Sive H. L. Grainger R. M. Harland R. M. (2007b). *Xenopus laevis* in vitro fertilization and natural mating methods. *CSH Protoc.* 2007:db.rot4737. 10.1101/pdb.prot4737 21357082

[B100] Slater P. G. Hayrapetian L. Lowery L. A. (2017). *Xenopus laevis* as a model system to study cytoskeletal dynamics during axon pathfinding. *Genesis* 55:e22994. 10.1002/dvg.22994 28095612 PMC5276718

[B101] Sojka S. Amin N. M. Gibbs D. Christine K. S. Charpentier M. S. Conlon F. L. (2014). Congenital heart disease protein 5 associates with CASZ1 to maintain myocardial tissue integrity. *Development* 141 3040–3049. 10.1242/dev.106518 24993940 PMC4197678

[B102] Stec I. Wright T. J. van Ommen G. J. de Boer P. A. van Haeringen A. Moorman A. F. (1998). WHSC1, a 90 kb SET domain-containing gene, expressed in early development and homologous to a *Drosophila* dysmorphy gene maps in the Wolf-Hirschhorn syndrome critical region and is fused to IgH in t(4;14) multiple myeloma. *Hum. Mol. Genet.* 7 1071–1082. 10.1093/hmg/7.7.1071 9618163

[B103] Sun J. Li R. (2010). Human negative elongation factor activates transcription and regulates alternative transcription initiation. *J. Biol. Chem.* 285 6443–6452. 10.1074/jbc.M109.084285 20028984 PMC2825440

[B104] Szabó A. Melchionda M. Nastasi G. Woods M. L. Campo S. Perris R. (2016). In vivo confinement promotes collective migration of neural crest cells. *J. Cell Biol.* 213 543–555. 10.1083/jcb.201602083 27241911 PMC4896058

[B105] Tahir R. Kennedy A. Elsea S. H. Dickinson A. J. (2014). Retinoic acid induced-1 (Rai1) regulates craniofacial and brain development in *Xenopus*. *Mech. Dev.* 133 91–104. 10.1016/j.mod.2014.05.004 24878353

[B106] Tandon P. Conlon F. Furlow J. D. Horb M. E. (2017). Expanding the genetic toolkit in *Xenopus*: approaches and opportunities for human disease modeling. *Dev. Biol.* 426 325–335. 10.1016/j.ydbio.2016.04.009 27109192 PMC5074924

[B107] Tandon P. Showell C. Christine K. Conlon F. L. (2012). Morpholino injection in *Xenopus*. *Methods Mol. Biol.* 843 29–46. 10.1007/978-1-61779-523-7_4 22222519 PMC3640826

[B108] Theveneau E. Mayor R. (2011). Beads on the run: beads as alternative tools for chemotaxis assays. *Methods Mol. Biol.* 769 449–460. 10.1007/978-1-61779-207-6_30 21748694

[B109] Theveneau E. Steventon B. Scarpa E. Garcia S. Trepat X. Streit A. (2013). Chase-and-run between adjacent cell populations promotes directional collective migration. *Nat. Cell Biol.* 15 763–772. 10.1038/ncb2772 23770678 PMC4910871

[B110] Toyokawa G. Cho H.-S. Masuda K. Yamane Y. Yoshimatsu M. Hayami S. (2011). Histone lysine methyltransferase Wolf-Hirschhorn syndrome candidate 1 is involved in human carcinogenesis through regulation of the Wnt pathway. *Neoplasia* 13 887–898. 22028615 10.1593/neo.11048 PMC3201566

[B111] Wallmeier J. Shiratori H. Dougherty G. W. Edelbusch C. Hjeij R. Loges N. T. (2016). TTC25 deficiency results in defects of the outer dynein arm docking machinery and primary ciliary dyskinesia with left-right body asymmetry randomization. *Am. J. Hum. Genet.* 99 460–469. 10.1016/j.ajhg.2016.06.014 27486780 PMC4974089

[B112] Wang F. Shi Z. Cui Y. Guo X. Shi Y.-B. Chen Y. (2015). Targeted gene disruption in *Xenopus laevis* using CRISPR/Cas9. *Cell Biosci.* 5:15. 10.1186/s13578-015-0006-1 PMC4403895 25897376

[B113] Willsey H. R. Walentek P. Exner C. R. T. Xu Y. Lane A. B. Harland R. M. (2018). Katanin-like protein Katnal2 is required for ciliogenesis and brain development in *Xenopus* embryos. *Dev. Biol.* 442 276–287. 10.1016/j.ydbio.2018.08.002 30096282 PMC6143417

[B114] Yamada-Okabe T. Imamura K. Kawaguchi N. Sakai H. Yamashita M. Matsumoto N. (2010). Functional characterization of the zebrafish WHSC1-related gene, a homolog of human NSD2. *Biochem. Biophys. Res. Commun.* 402 335–339. 10.1016/j.bbrc.2010.10.027 20946879

[B115] Yung T. M. C. Narita T. Komori T. Yamaguchi Y. Handa H. (2009). Cellular dynamics of the negative transcription elongation factor NELF. *Exp. Cell Res.* 315 1693–1705. 10.1016/j.yexcr.2009.02.013 19245807

[B116] Zahn N. Levin M. Adams D. S. (2017). The Zahn drawings: new illustrations of Xenopus embryo and tadpole stages for studies of craniofacial development. *Development* 144 2708–2713. 10.1242/dev.151308 28765211 PMC5560046

[B117] Zhang X.-L. Huang C.-X. Zhang J. Inoue A. Zeng S.-E. Xiao S.-J. (2013). CtBP1 is involved in epithelial-mesenchymal transition and is a potential therapeutic target for hepatocellular carcinoma. *Oncol. Rep.* 30 809–814. 10.3892/or.2013.2537 23756565

[B118] Zhang Z. Song Y. Zhao X. Zhang X. Fermin C. Chen Y. (2002). Rescue of cleft palate in Msx1-deficient mice by transgenic Bmp4 reveals a network of BMP and Shh signaling in the regulation of mammalian palatogenesis. *Development* 129 4135–4146. 12163415 10.1242/dev.129.17.4135

[B119] Zollino M. Orteschi D. Ruiter M. Pfundt R. Steindl K. Cafiero C. (2014). Unusual 4p16.3 deletions suggest an additional chromosome region for the Wolf-Hirschhorn syndrome-associated seizures disorder. *Epilepsia* 55 849–857. 10.1111/epi.12617 24738919

